# Study of Concrete under Combined Action of Aggressive Environment and Long-Term Loading

**DOI:** 10.3390/ma14216612

**Published:** 2021-11-03

**Authors:** Yaroslav Blikharskyy, Jacek Selejdak, Nadiia Kopiika, Rostyslav Vashkevych

**Affiliations:** 1 Department of Highways and Bridges, Lviv Polytechnic National University, 12 st. S. Bandera, 79013 Lviv, Ukraine; Yaroslav.Z.Blikharskyy@lpnu.ua; 2 Faculty of Civil Engineering, Czestochowa University of Technology, 69 st. Dabrowskiego, 42-201 Czestochowa, Poland; 3 Department of Building Constructions and Bridges, Lviv Polytechnic National University, 12 st. S. Bandera, 79013 Lviv, Ukraine; kopijka.nadija.1999@gmail.com (N.K.); rostyslav.v.vashkevych@lpnu.ua (R.V.)

**Keywords:** corrosion damages, concrete corrosion, material properties, corrosion in RC constructions

## Abstract

A significant part of reinforced concrete structures is subjected to intensive environmental impact during operation. This can cause local destruction and failure of buildings if obligatory measures are not taken to protect them from corrosion. This is especially true for industrial buildings, where the environment could be contaminated with aggressive products or waste. An important issue is the development of methods for calculating the load-bearing capacity and serviceability of reinforced concrete structures with corrosion damage. The main reason for this is the necessity to determine the durability and reliability of buildings and structures and the estimation of their safe operation time. As corrosion damages of concrete are a critical issue, more detailed experimental studies are needed. This paper presents experimental studies of concrete prisms under the simultaneous action of an aggressive environment and a constant level of compressive force. In total, 32 prisms under different loading conditions and in different aggressive medium were tested. Samples were divided in series, for which different load levels were chosen ($0.25f_{ck}$, $0.35f_{ck}$, $0.45f_{ck}$). Additionally, control samples in the air and immersed in water were tested. During the experiment, different parameters were monitored and recorded: decrease of cross-sectional size, the temperature and environmental humidity. Results of the study showed that destruction occurred due to the presence of corrosion damages of concrete and a reduction of the cross-sectional area. The stresses in the concrete at the destruction stage were less than the value of the prism strength by 10–12%. It was established that along the contour of the section, there is a partially degraded layer of concrete of 1.5–3.7 mm thickness, with corrosion microcracks and corrosion products. Additionally, experimental and theoretical diagrams of concrete with corrosion damages were obtained and compared. The ultimate deformations of concrete with corrosion damage, which correspond to the prismatic strength of concrete, in comparison with undamaged concrete were lower by 11–18%. Therefore, the concrete strength is decreased during exploitation under loading in an aggressive environment, which needs to be taken into account during calculations.

## 1. Introduction and Literature Review

Intensive environmental impact is an important problem for concrete and reinforced concrete structures which are currently operated. This effect could be the reason for local destruction and failure of buildings [1,2,3,4,5]. In order to avoid this, necessary measures should be taken to protect concrete from corrosion [6,7]. Therefore, during the further operation, there a need for strengthening and restoration of such structures [8,9,10]. This is especially true for industrial buildings, where the medium (liquid and gaseous), which contacts with the surface of building structures, could be contaminated with aggressive products or industrial waste [11,12,13]. Therefore, complete understanding of corrosion processes and a reliable assessment of their influence on residual bearing capacities of structures have recently become topical issues of scientific research.

Physical and chemical processes during corrosion are considered in a number of recent studies [14,15,16,17,18]. Thus, there is a general opinion that the main cause of the concrete destruction is the osmotic pressure inside the closed cells, which causes the destruction of cement stone. However, the estimation of conditions which cause osmotic pressure in the cement stone and their possible role in corrosion destruction is realizable only on the basis of experiments for particular conditions. Studies of the role of magnesium compounds in the development of osmotic processes [19] have shown that concrete with increased density of cement mortar shows some signs of semi-permeability. When magnesium salts interact with calcium hydroxide, amorphous magnesium hydroxide is deposited in the pores of the concrete, which enhances the semi-permeability effect.

One of the parameters of the concrete corrosion resistance is its water resistance, which can be increased, both by design measures and with special additives in the concrete [20]. It is established that the water resistance of concrete depends on the number and type of pores, the ratio of raw materials, the selection of the composition of the concrete mixture and the concreting technique. Additionally, it is proposed to increase the water resistance of concrete by the addition of ferric chloride, as one of the concrete components. The addition of ferric chloride not only increases the water resistance of concrete, but also accelerates the process of the strength increase by 1.2–1.3 times. However, as it is generally known, the presence of chlorides in reinforced concrete could cause the reinforcement corrosion.

As the alternative to cementitious binder concretes, new types of acid-resistant concretes have been developed and studied. As the result of the research in [21], the possibility of using complex binder on the basis of sodium glass and perlite for acid-resistant concrete was theoretically substantiated and experimentally established. Acid resistance and the strength of concrete increase if ground quartz is added to the mixture.

Many studies concern the effect of corrosion on modified concrete [22,23,24,25]. The kinetic dependence of corrosion processes over time was established on the example of concrete with polymer silicate binder, which is considered to be acid-resistant. This dependence allows to assess the level of the aggressiveness of the environment and to predict the depth of ions’ penetration into concrete. The chemical stability of the developed polymer concretes in different media (organic acids, alkalis) was studied. It was found that there is no active chemical interaction between the material and the aggressive media, and the decrease in physical and mechanical properties is limited and weakens over time. According to the authors of [22,23,24,25], this enables to obtain reliable protection of building structures by introduction of the optimal number of recommended modifiers and the application of developed techniques. However, along with the advantages, there is a disadvantage—the cost of acid-resistant concrete increases significantly, sometimes by several times compared to cement concrete.

Additives, which modify the concrete structure and properties, are of considerable interest as the means to increase its corrosion resistance. The authors of [26,27,28,29] considered the effectiveness of modifiers with hydrophobic, hydrophobic-structural, air-absorbing and plasticizing properties and complex modifiers for different purposes. In addition, the possibility to increase the frost and corrosion resistance of concrete in the conditions of capillary rise and precipitation of salt solutions was shown, as well as their long-term continuous action. For tar concrete, an effective way to increase corrosion resistance could be the introduction of modifying additives: primary waste from the production of polyvinyl chloride, as well as complex additives—polymers and active dispersed fillers. The introduction of tar additives allows to increase the adhesion and activation energy of concrete. Additionally, they can expand the range of plasticity and ensure elastic properties in the conditions of negative temperatures. The issue of possible ways to increase corrosion resistance of concrete structures is reviewed and summarized in the work of Coppola [30]. Thorough analysis of recent findings brought the author [30] to the conclusion that the durability of newly built concrete structures can be ensured with proper mix design and construction details. However, in cases of existing structures or severely aggressive environments, corrosion inhibitors, surface treatments and cathodic protection are more applicable [30].

An important issue is the development of methods for calculating the load-bearing capacity and serviceability of reinforced concrete structures with corrosion damages. The main reason is the necessity of determination of the durability and reliability of buildings and structures, as well as the assessment of their safe operation time. Most researchers [29,30,31,32,33,34,35,36,37,38,39,40,41,42,43,44,45,46] agree that quantitative and qualitative evaluation of the residual bearing capacity of damaged material should include consideration of specifics of physical and chemical corrosion mechanisms. This problem was thoroughly discussed in a number of previous research works. The main fundamental principles of corrosion processes in concrete and possible ways of its evaluation were described by Hobbs [31], Beeby [32] and Weyers [33]. Thus, according to Hobbs [31], concrete deterioration could be described by the process of ion ingress, and is defined by chemical alkali–silica reactions, freeze–thaw attack, carbonation and external and internal chemical attack. Weyers [33] describes the main stages of the concrete corrosion and proposes evaluation models, which include factors of structure deterioration and could increase its maintenance.

As Webster [34] states, the assessment of corrosion-damaged concrete structures anticipates the necessity to take into account variability and uncertainty in the input parameters and analyze each particular structural situation as a unique process. Webster [34] studied mechanisms of chloride-induced corrosion and their influence on bond, flexural and shear strength of structures. It was indicated that reliable assessment of corrosion mechanisms in concrete requires a great amount of input experimental information, which could be obtained either from field measurements or laboratory modeling of corrosion processes [34,35].

Thus, in recent times, studies dedicated to qualitive and quantitative assessment of corrosion processes in concrete with the use of accelerated corrosion tests are widespread [36,37]. Generally speaking, during such tests, the effect of corrosion is evaluated according to values of material loss. An important advantage of such techniques is the possibility to monitor the progression of the corrosion current and measure the dynamics of the corrosion process [36]. For example, Ferreira et al. [36] simulated the behavior of reinforced concrete in the marine environment with the use of the electrochemical method for accelerated modeling of an aggressive chloride environment. It is also important to note the work of Ahmad [37], in which the method for evaluation of the corrosion resistivity of concrete is proposed on the basis of empirical correlation to reinforcement corrosion rate according to the measured corrosion current density.

Electrical measurement methods of chemical processes in concrete were also conducted by Kusak et al. [38,39,40], Cabeza [41] and Macdonald [42].

Namely, in [41,42], fundamental principles of the spectroscopy method for microstructural analysis of concrete are investigated, which provide the possibility to identify the component chemical composition of concrete material, subjected to aggressive impact. Another method for analysis of corroded concrete structures is proposed in [43,44,45]. For instance, Zaki et al. [43] applied the AE-technique for experimental investigation of fracture processes in the corroded beam specimen under flexural load. Authors in [44,45] propose the methodology for assessment of damage intensity of the RC structures on the basis of the AE amplitude distribution.

It could be summarized that quantitative evaluation of concrete corrosion in recent research studies is circumscribed by analysis of experimental data, based on mass or cross-section loss during the corrosion process. However, there are the number of issues of the reinforced concrete structures with corrosion damage of concrete, which are not sufficiently studied yet. Therefore, further investigation of this urgent issue and more detailed experimental studies of the effect of corrosion on concrete damage are required.

## 2. Materials and Methods

Experimental research included testing of prism samples, made of concrete with the following characteristics: Prisms were made of concrete with components ratio C:S:Gr = 1:1.01:2.28 and water-cement ratio W/C = 0.32. Additionally, the super-plasticizer “Chrysofluid” (Chryso, Les Moulineaux, France, ρ = 1.148 g/cm^3^) and air-absorbing additive “Chrysoair” (Chryso, Les Moulineaux, France, ρ = 1.03 g/cm^3^) were used. The dosage of the super-plasticizer was 1.2 kg for 100 kg of cement and 0.035 kg of the air-absorbing additive for 100 kg of cement.

The detailed information about the mixing composition of concrete is provided in Table 1.

Cement used in the experiment was of the M400 and M500 grades from the Mykolaiv cement plant (Mykolaiv cement plant, Mykolaiv, Lviv region, Ukraine). Detailed characteristics of the concretes used during the experiment are provided in Table 2.

**Figure 1:**
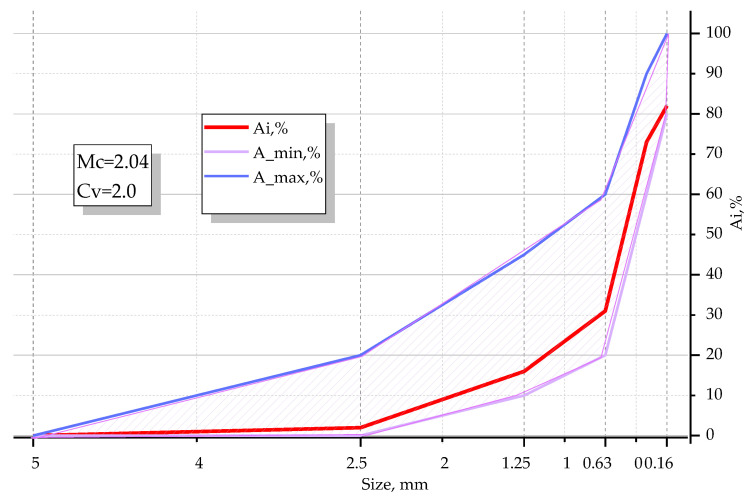
Granulometric curve for fine aggregates (Ai—total residues on a sieve of given cell size, Ai_max, Ai_min—maximum and minimum values of total residues).

Fine and coarse aggregates were tested with the use of the sieve analysis procedure. As the fine aggregate, quartz sand from Slavutych quarry of the Khmelnytsky region (Budquartzservis, Slavutych quarry, Khmelnytsky region, Ukraine) was used, without impurities and with a size modulus of M_c_ = 2.04 and coefficient of non-uniformity C_v_ = 2.0. The granulometric curve for fine aggregates is presented in Figure 1. 

**Figure 2:**
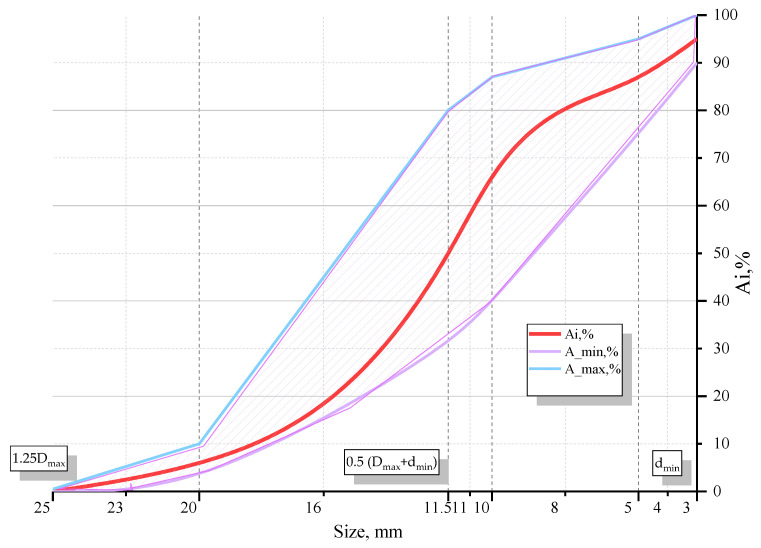
Granulometric curve for coarse aggregates (Ai—total residues on a sieve of given cell size, Ai_max, Ai_min—maximum and minimum values of total residues).

As the coarse aggregate, granite crushed stone from the Selishche quarry of the Rivne region (LLC “Selishchansky granite quarry”, Selische, Rivne region, Ukraine) was used. Maximum D_max_ and minimum d_min_ diameter of gravel grains were 20 and 3 mm, respectively. Fractional composition of coarse aggregates mostly contained 5..10 and 10..20 mm grains. The granulometric curve for coarse aggregates is presented in Figure 2.

The experimental study of concrete was performed by testing prism samples of 100 mm × 100 mm × 400 mm size according to regulations of DSTU B B.2.7–214: 2009, EN 206:2013.

**Figure 3:**
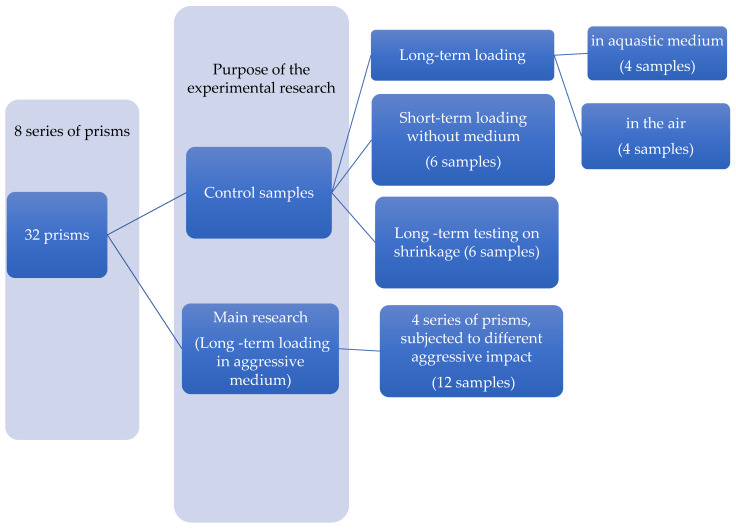
The number of samples for experimental research of concrete prisms.

Figure 3 contains information about the number of samples tested during the experiment and principles of their division into the series.

Samples were subjected to the action of an acid-liquid environment and axial compression in order to study the change of physical and mechanical properties of concrete under loading over time, taking into account corrosion damages of concrete.

In order to study the effect of stress on the development of corrosion processes in concrete over time, 4 series of prisms were loaded with different values of initial stresses, equal to $0.25f_{ck}$, $0.35f_{ck}$ and $0.45f_{ck}$. For series 1, the load level was equal to 0.25$f_{ck}$, for series 2 it was equal to 0.35$f_{ck}$, for series 3 the load level was 0.45$f_{ck}$ and for series 4 it was 0.35$f_{ck}$. Here, for series 1–3, the compressive strength was $f_{ck}$= 41 MPa and for series 4 it was equal to $f_{ck}$= 53.6 MPa. According to the accepted load levels of 0.25, 0.35 and 0.45, initial stresses of 10.25, 14.35 and 18.45 MPa were created in the prisms by means of strands with nuts. 

**Figure 4:**
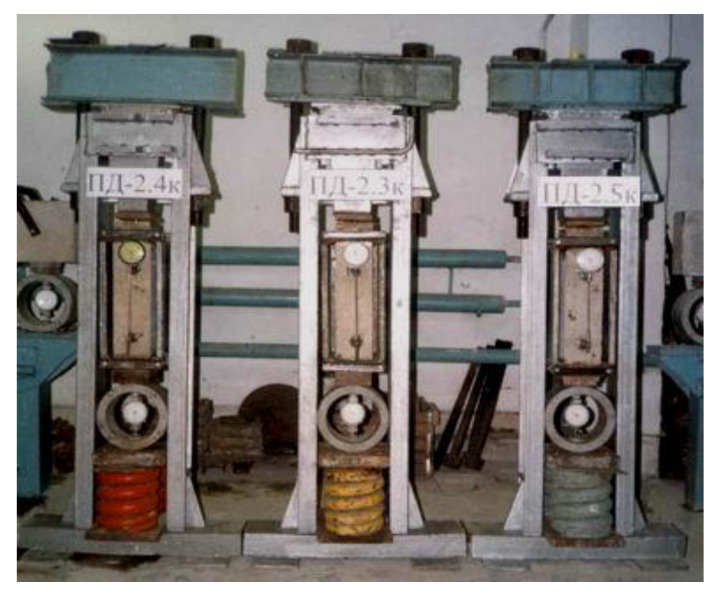
Prisms subjected to long-term loading before the experiment.

**Figure 5:**
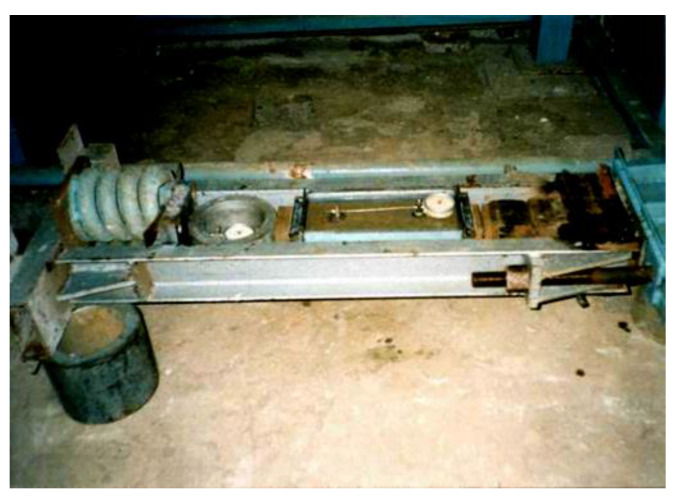
The power stand for experimental testing of prisms in aggressive medium.

Series 1–4 of prisms belonged to the main part of the research (see Figure 3). For each level of loading, a minimum of three prisms of one concrete series were loaded for one series of the experiment (Figure 4 and Figure 5). The prisms were tested by applying the following loading conditions and aggressive impact:

(a) Only the central compressive force without aggressive medium,
(b) Simultaneous influence of compressive force and aquatic medium,
(c) Simultaneous influence of compressive force and aggressive environment.

The following symbols were used for the marking of prisms: P—prism, and L—tested for long-term loading (see Table 3). The marking included two numbers, the first of which indicates the series of prisms (1, 2, 3, 4), and the second indicates the sequence number of the sample in this series (1, 2, 3, 4, 5). The letter “c” shows that the prism was subjected to aggressive impact (corrosion).

In order to take into account the whole range of various factors, which have an influence on the destruction process, series 5–8 of control samples were tested:

- Series 5 contained 4 prisms, tested in the air, subjected to long-term loading.
- Series 6 contained 6 prisms, tested in the air, subjected to short-term loading.
- Series 7 contained 6 prisms, tested on shrinkage.
- Series 8 contained 4 prisms, tested in aquatic medium, subjected to long-term loading.

In total, 32 prisms were tested under different conditions (Figure 3).

Concrete prisms were tested according to the developed technique on the special stand. The constant value of compressive force was provided with the use of powerful springs.

During the first 24 h after concreting, the samples were covered with plastic wrap to prevent heat loss during cement hydration. After that, the strength setting was continued at the external temperature of 20 °C and constant humidification. Concrete samples were released from the formwork on the 3rd–4th day after concreting.

In order to determine the initial physical and mechanical characteristics of the studied material, control samples were investigated (see Figure 3). Thus, 3 prisms of each concrete type (in total 6 samples) were tested under short-term loading without medium (series 6), and 4 samples were tested under long-term loading in the air (series 5).

Micro-indicators for the determination of shrinkage deformations were fixed on 6 prisms, that were not subjected to loading (series 7).

In order to assess the water resistivity of concrete, 4 samples were tested in aquatic medium under long-term loading (series 8).

Simultaneously with the loading of concrete prisms, concrete cubes of 100 mm × 100 mm × 100 mm size were immersed in the aggressive environment of the same concentration in special containers of 120 mm× 120 mm× 120 mm size, in cubic form. The purpose of this experiment was observation of the corrosion depth and strength characteristics of cube samples.

As the aggressive medium, the solution of sulfuric acid H_2_SO_4_ with 10% concentration was used. Such concentration of the acid medium enabled the accelerated modeling of the aggressive environment impact and the assessment of the structure behavior in particular conditions. By type, these are corrosion processes that develop in concrete under the action of water containing chemicals that react with the components of cement stone and belong to highly aggressive medium. A similar environment takes place in some chemical plants, galvanic shops, chimneys of thermal power stations and reinforced concrete structures of scrubbers in emergency situations. In addition, the creation of such aggressive environment allows to simulate the effect of an aggressive environment and the simultaneous action of the load in a relatively short period of time.

**Figure 6:**
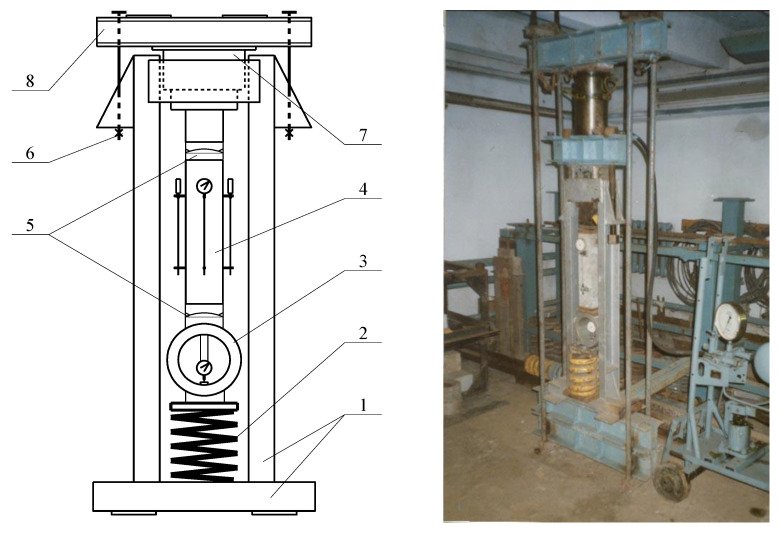
Scheme and general view of the power stand for loading prisms and general view of the power stand for loading of concrete prisms with long-term loading: 1—frame of the power stand; 2—springs; 3—ring dynamometer; 4—prism; 5—spherical hinge; 6—fixing strands; 7—heel for load transfer; 8—distribution, traverse.

Prism samples were loaded on a specially made power stand (Figure 6) to a given level of load; with the help of strands, samples were fixed at this loading level. During loading, the prism was centered with the use of four clock-type indicators with measurement accuracy of 0.01 mm. Indicators were placed on the faces of the prism with a measurement base of 200 mm

Next, special containers with aggressive medium were fixed on experimental samples of concrete prisms, and the joints were carefully sealed. 

Before the beginning of the experiments, the geometric dimensions, the base for concrete deformation measurement and the mass of the samples were carefully recorded using a caliper and electronic scales. During the research, these values were constantly monitored. Additionally, during the experiment, the temperature, ambient humidity and acid concentration were constantly monitored. When the acid concentration changed by more than 1%, the acid solution was replaced. 

It should be noted that the age of the concrete prisms from the moment of concreting until the beginning of the testing was 520 days. During this time, the main part of shrinkage deformations occurred, and the shrinkage processes of concrete were almost stabilized, which could be confirmed by the readings of devices on prisms studied for shrinkage (series 7).

Measurements of longitudinal deformations of concrete were performed daily, while cross-sectional dimensions and the weight of samples were controlled every 10 days. Besides the measurement of deformations after the destruction of prisms, X-ray phase analysis of cement stone and study of the concrete microstructure were also performed in order to detect corrosion products and changes in the concrete structure. X-ray phase analysis was performed on an X-ray machine, DRON-2. For X-ray phase analysis, samples were taken from each sample from the surface and from the depth of concrete prisms. 

**Figure 7:**
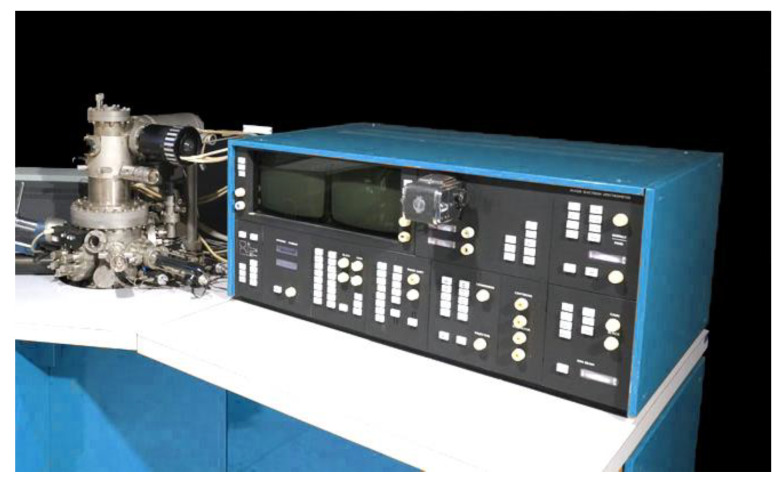
Electron microscope TESLA BC—300.

The concrete microstructure (TESLA, Prague, Czech Republic) was studied with the use of the raster electron microscope TESLA BC—300 (Figure 7).

Samples were taken in the form of specimens with dimensions of 10 mm × 10 mm × 15 mm so that they included the outer and inner layers of the studied material. Therefore, it was possible to trace the changes in the microstructure of concrete in different areas. In order to obtain the clear idea of the concrete microstructure, the images of the samples were magnified 1000 times and fixed on photographic film. In order to analyze the microstructure of concrete, three photographs were taken: the first at the surface, the second at the distance of 4–6 mm and the third at the distance of 10–15 mm from the surface.

## 3. Results and Discussion

### 3.1. Experimental Testing of the Concrete Prisms in the Aggressive Medium 

According to the accepted research method of the concrete deformability under long-time impact of aggressive medium and compressive forces, four series of concrete prisms were tested. For series 1, the load level was equal to 0.25$f_{ck}$, for series 2 it was equal to 0.35$f_{ck}$, for series 3 the load level was 0.45$f_{ck}$ and for series 4 it was 0.35$f_{ck}$. Here, for series 1–3, the compressive strength was $f_{ck}$= 41 MPa and for series 4 it was equal to $f_{ck}$= 53.6 MPa. The techniques of the experimental research and marking of the samples are presented in Section 2. 

During the experiment, according to the accepted technique, the sizes of the cross-section and changes of deformations of concrete were measured. Destruction processes in concrete, which were observed during the test, were caused by concrete corrosion. For better understanding of deterioration processes, which are the issue of this study, it is necessary to provide a detailed explanation on the main mechanisms of concrete destruction. In general, concrete corrosion could be identified with chemical reactions of acid with components of cement stone, containing calcium.

The chemical reaction could be described by Equation (1):

Ca(OH)_2_+ H_2_SO_4_→CaSO_4_·2H_2_O. (1)

CaSO_4_·2H_2_O in Equation (1) is gypsum. As gypsum is the salt of sulfuric acid, it simultaneously proceeds to the sulfate corrosion (see Equation (2)):

3CaO·Al_2_O_3_·12H_2_O+3(CaSO_4_·2H_2_O)+13H_2_O→3CaO·Al_2_O_3_·3CaSO_4_·32H_2_O. (2)

As the result of this reaction (2), the crystals of ettringite or “cement bacillus” are formed (3CaO·Al_2_O_3_·CaSO_4_·32H_2_O). Under conditions of repeated wetting and drying, ettringite crystals tend to absorb additional moisture, which causes their expansion. Expanded crystals become the reason for localized crystallization pressure and internal stresses. After that, due to macroscopic expansion of the hardened material, the destruction of concrete proceeds.

Together with gypsum, the components of concrete-quartz sand and granite rubble, which are practically acid-resistant materials, precipitate. This also causes the material loss and decrease of the size of the cross-section.

Equations (1) and (2) describe the formation of ettringite crystals. It is important to note that the corrosion process envies different chemical processes between the components, so that the destruction of the structural element is the result of a combination of different chemical processes. 

Simultaneously with ettringite formation, the acid aggressive medium H_2_SO_4_ could react with calcium components, Ca(OH)_2_, which results in a chemical reaction of neutralization. Therefore, C-S-H phases are destructed, and the concrete material is deteriorated, which causes the loss of mass and decrease of dimensions.

Another crucial destruction mechanism is the carbonation, which progresses in the following way: The carbon dioxide enters the concrete pores from the atmosphere and causes the carbonation reaction with interstitial water. After that, calcium hydroxide “hydrated lime” is converted. As the calcium hydroxide provides high values of pH of concrete, its destruction lowers the pH of concrete stone to the values up to 8–9 and also degrades the passivation of the reinforcement [46].

Important issues in concrete corrosion are reactions of alkalis in concrete with aggregates. These processes are generally called alkali reactions and cause tension stresses due to components’ expansion, after which swelling and cracks appear [46].

Among chemical causes of concrete destruction are also internal and external sulphate reactions [46]. The process of concrete deterioration in this case is determined by precipitation of secondary sulphates and chemo-mechanical deterioration. As a result, strength is greatly reduced and cracking begins. Finally, the cementitious material can be almost destroyed.

Through the experimental research, diligent observations and a detailed analysis of the specifics of the deterioration process were made. During the whole test period, white precipitate was constantly released on the samples, which were immersed in the sulfuric acid.

**Figure 8:**
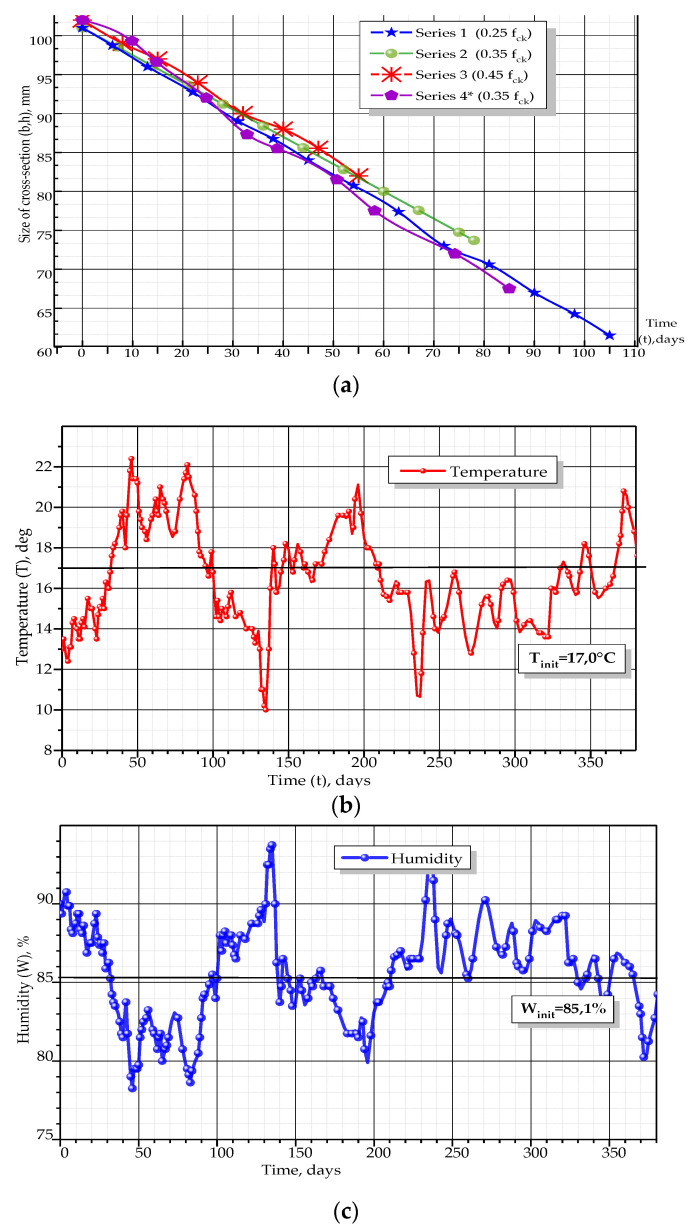
Recordings made during the experiment: (**a**) changes in cross-section sizes for prisms, (**b**) changes of temperature and (**c**) changes of humidity.

This substance is the product of concrete corrosion, which was formed due to chemical reactions (see Equations (1) and (2)). The white sediment was gypsum, which, as a result of reaction (2), formed the crystals of ettringite or “cement bacillus”. At the same time, the cross-sectional dimensions of the concrete samples constantly decreased (see Figure 8a).

In the described experiments, the cross-sectional dimensions were recorded by direct measurement with the caliper with an accuracy of 0.05 mm.

Experimental studies have shown that the sizes of the cross-section (width b and height h) decreased during the test, almost linearly (see Figure 8a). At the same time, the cross-sectional area decreased in quadratic dependence. During the first days of the experiment, the intensity of the size reduction was slowed down, which is associated with the sealing of concrete pores with the initial corrosion products. Next, the corrosion products were dissolved, and the rate of size changes was stabilized. Additionally, the difference in the residence time for prisms was noted, which increased at decreased initial stress levels. 

Parameters of the aggressive medium could have a significant impact on the corrosion process, as they may act as initiators or inhibitors of the corrosion rate. On the other hand, changes in the moisture content or its temperature can be considered as indicators of the intensity of chemical reactions. Therefore, during the experiment, the temperature and environmental humidity were constantly monitored (Figure 8b,c). Recording of the temperature and air humidity began 250 days before the loading of samples was started in order to track its changes while the samples were cured without loading. Temperature was on average 17 °C and was maintained the same for aggressive medium and air (see Figure 8b). Before the loading of samples was started (days 0–250 in Figure 8b), deviations amounted about ±5 °C. During the loading period, these values were even smaller (±3 °C). Such changes indicate chemical processes, which proceed in concrete stone and are admissible for the engineering experiment.

The average air humidity was 85.1% and was almost equal to its initial level (see Figure 8c). During the curing period, separate deviations were recorded with the magnitude of up to ±10%, which indicates the hydration process in concrete during curing. During the loading period (days 250–380 in Figure 8c), deviations of humidity did not exceed ±5%, which is within the admissible level of accuracy

Thus, it could be assumed that temperature and humidity did not have a critical influence on the studied deterioration process.

It should be noted that during the tests, the measured total deformations of concrete included compressive deformations, creep deformations of concrete and shrinkage–swelling deformations (depending on the presence of prisms in the dry environment or in liquid medium). Further analysis will be more representative in coordinates of stress values, which corresponded to each loading level. Values of stresses were determined by dividing the load, N_ult_, applied by the power stand on the residual cross-sectional area, *S* (see Table 3).

The constant decreasing of the cross-section of prisms led to an increase of the stress level, $\sigma_{c}$, and ratio, $\sigma_{c}/f_{ck}$, because the value of compressive force was the same during the experiment. 

**Figure 9:**
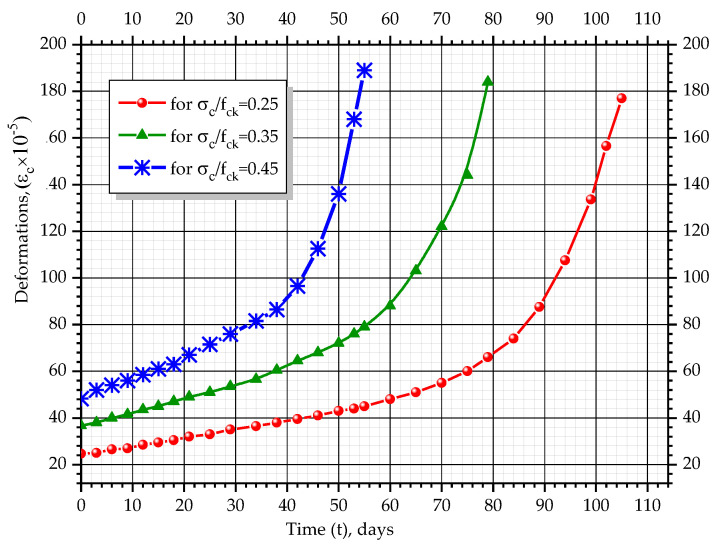
Changes in concrete deformation for tested prisms.

According to the measured values of longitudinal deformations of the concrete and the determined values of stresses, graphs, which show changes of deformations over time, were constructed (*ε_c_ − t*). Additionally, graphs showing changes of stress level over time ($\sigma_{c}/f_{ck}$ − *t*) and graphs of coordinates (*σ_c_* − *ε_c_*) were composed. The graphs show the average values of corresponding parameters for prism-twins (Figure 9).

All prisms with different stress levels are characterized by an almost linear increase in concrete deformations over time at the initial stages of the experiment. For prisms with a stress level equal to $\sigma_{c}/f_{ck}$= 0.25, this tendency is observed for a period of 70 days with a deformation increment of 27 × 10^−5^, for a stress level $\sigma_{c}/f_{ck}$= 0.35, for the 60 day period with a deformation increment of 46 × 10^−5^ and for $\sigma_{c}/f_{ck}$= 0.45, until day 40 of the experiment with a deformation increment of 43 × 10^−5^. In the next stages of the experiment, the deformation changes were non-linear until the destruction of the samples occurred. At the final stage, the deformation was equal to 177–192 × 10^−5^ for all the prisms with different initial stress levels. The character of stress level changes, $\sigma_{c}/f_{ck}$, over time is similar. The non-linear character of deformation changes could be explained by the fact that during the experiment, the cross-section of samples changes according to quadratic dependency. This leads to increments in stresses in concrete, *σ_c_*, and ratio, $\sigma_{c}/f_{ck}$. With the growth of the stress level, $\sigma_{c}/f_{ck}$, creep deformations considerably increased, as well as the plastic part of the deformations due to the action of compressive forces, which are part of total concrete deformations.

**Figure 10:**
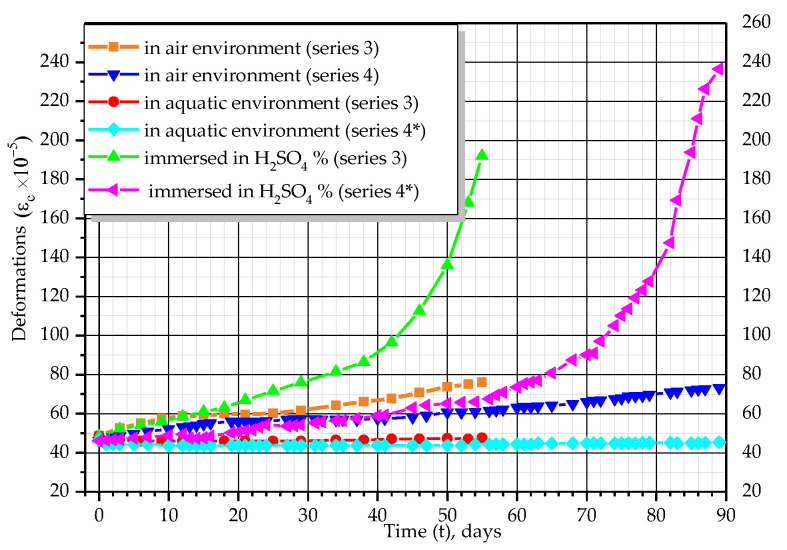
Changes of average deformations of concrete over time.

**Figure 11:**
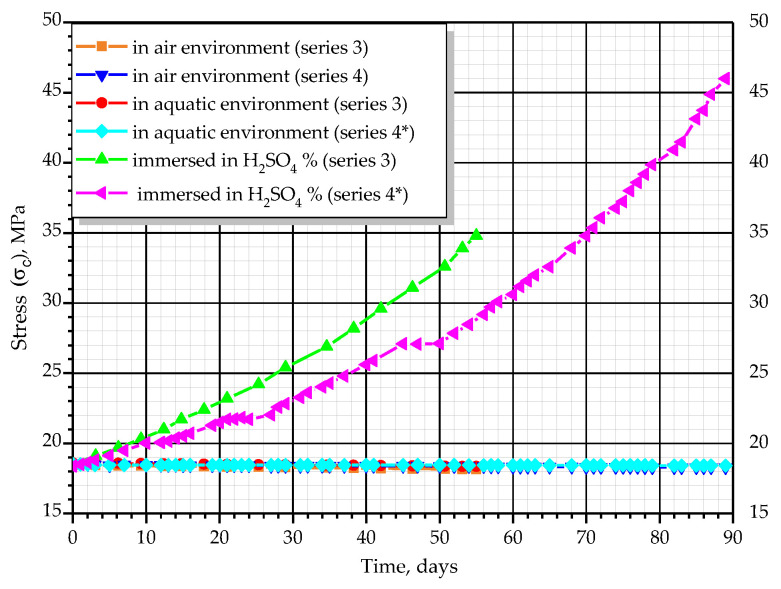
Changes of average stresses of concrete over time.

**Figure 12:**
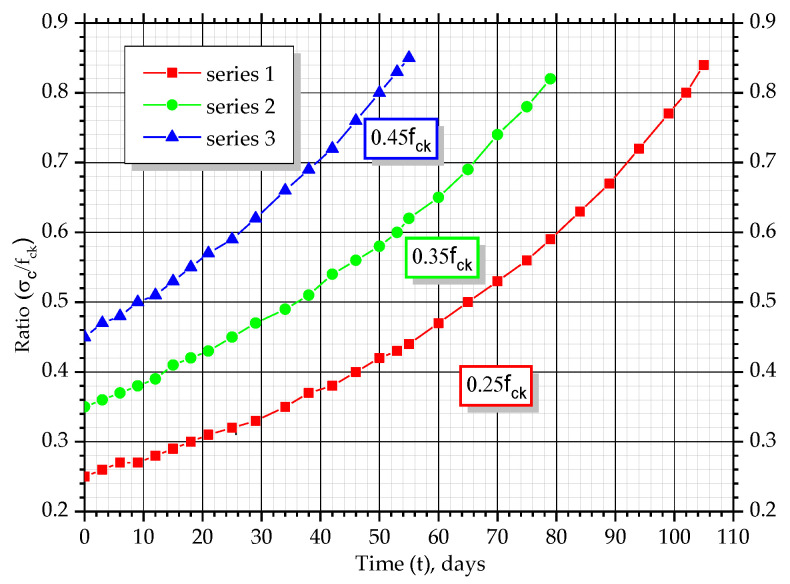
Changes of average values of *σ_c_*/*f_ck_* over time.

**Figure 13:**
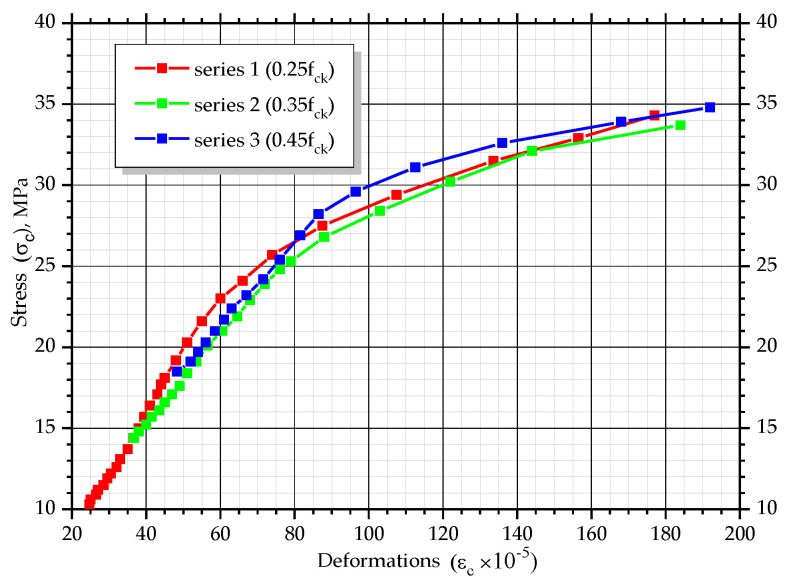
Average dependencies, *σ_c_* − *ε_c_*, in acid H_2_SO_4_.

As can be seen from the graphs in Figure 10, Figure 11, Figure 12 and Figure 13, the relationship between stresses, *σ_c_*, and deformations, *ε_b_*, was curvilinear for almost all prisms. Linear dependence was observed only in the initial stages of testing. 

For prisms of series 3 and 4 with an initial stress level equal to $\sigma_{c}/f_{ck}$*=* 0.45 and $\sigma_{c}/f_{ck}$*=* 0.35, which were tested in water, almost no increase in deformations was observed. Fifty days after the beginning of the experiment, deformations reached 5.0–7.4 × 10^−5^, whereas on the 89th day they decreased and were equal to 3.5–4.5 × 10^−5^ for series 3 and 4.2–5.8 × 10^−5^ and 2.5–3.0 × 10^−5^ for series 4, respectively. In prisms tested in the air and immersed in water, the stresses did not change. 

In the prisms on which the shrinkage of concrete was studied, practically no increase in deformations was recorded. This is due to the significant age of concrete prisms. Deformations ranged from 5 × 10^−5^ depending on changes in temperature and humidity.

**Figure 14:**
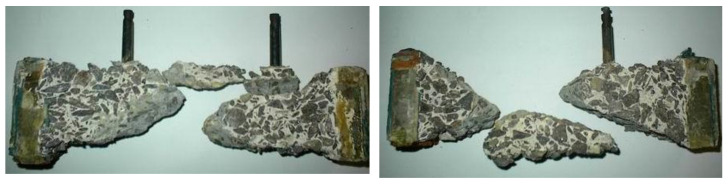
The character of the typical destruction of prism samples due to the corrosion of concrete.

The destruction of prisms tested in sulfuric acid occurred due to the decrease of the cross-sectional size and the increase of stresses in concrete under constant load. Destruction occurred on average 106 days after the moment of immersion in the aggressive environment for series 1, 79 days after immersion for series 2, 56 days for series 3 and 89 days for series 4. Destruction of all prisms took place along the oblique planes with the formation of cut pyramids (Figure 14). At the destruction stage, the cross-section area decreased on average by 63–65% for series 1, by 46–50% for series 2, by 34–37% for series 3 and by 49–51% for series 4.

The experimental values of the strength of prisms were compared with the calculated values according to the valid normative standards, taking into account the increase of the flexibility of prisms due to the reduction of the cross-sectional size (Table 3).

The theoretical values of the ultimate load, which could be perceived by the prismatic sample, were calculated with the use of the modified formula from Eurocode 2 (see Equation (3)):

N_ult_ = N_Rd_ = f_cd_ × A_c_, (3)

where f_cd_ is the design value of concrete strength, and A_c_ = S is the actual cross-section area.

In order to represent changes in concrete strength due to corrosion, experimental and theoretical values of the ratio N_ult_/S are provided in Table 4. Instrumental and total measurement errors are also shown in Table 4.

Analysis of the results of experimental studies shows that the destruction of concrete prisms in sulfuric acid was due to the corrosion of concrete. Corrosion processes caused the linear decrease in the cross-sectional size of prisms over time. The cross-sectional area of the experimental samples decreased according to quadratic dependency. The decrease in the cross-sectional area caused an increase in stresses in the concrete and in flexibility, which led to the destruction of concrete prisms. The destruction took place at a stress level lower than the initial strength of concrete. The reason is the decrease in the strength of concrete with corrosion damages during long-term loading, which could be taken into account by the coefficient of working conditions of concrete in an aggressive environment, γ_bc_. For the concrete prisms tested in the air and water, no destruction occurred, and the stress level in concrete remained constant. A slight increase in deformations of concrete due to shrinkage or swelling and creep was observed. In concrete prisms immersed in water, the deformations of concrete creep were balanced by swelling deformations, and therefore total deformations remained unchanged.

The analysis also shows that the methodology of valid norms in all cases does not allow to determine with sufficient accuracy the strength of compressed concrete elements with corrosion damages. The difference between theoretical and experimental values was about 12.3% to 19.5% (see Table 3). In general, theoretical values were higher than experimental ones.

### 3.2. Study of the Microstructure and Mineral Composition of Concrete after Exposure to Aggressive Environment 

**Figure 15:**
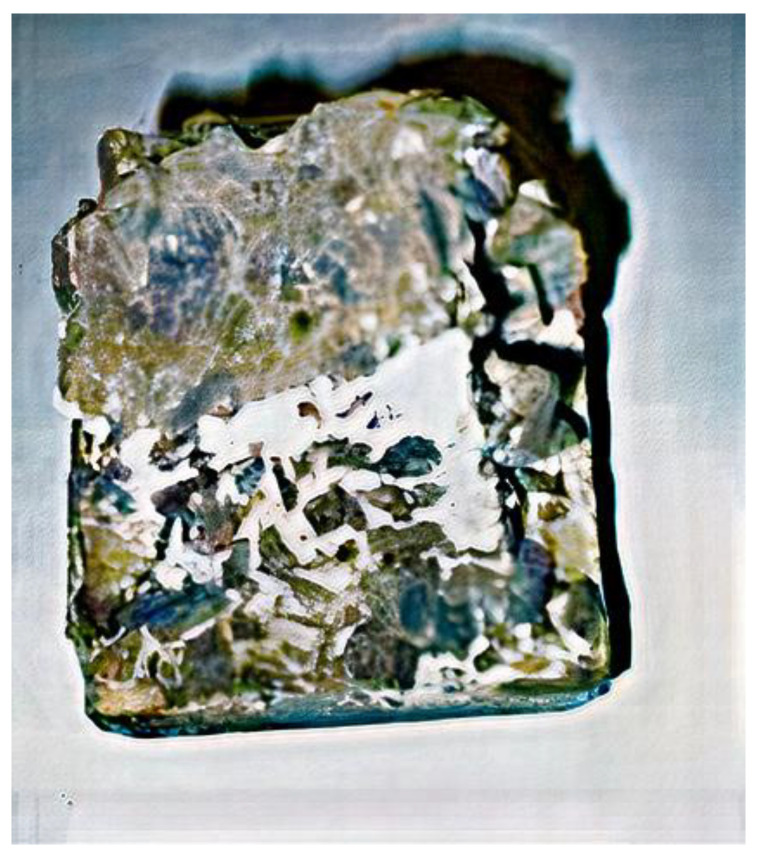
The cut of the sample after destruction in acid H_2_SO_4_.

**Figure 16:**
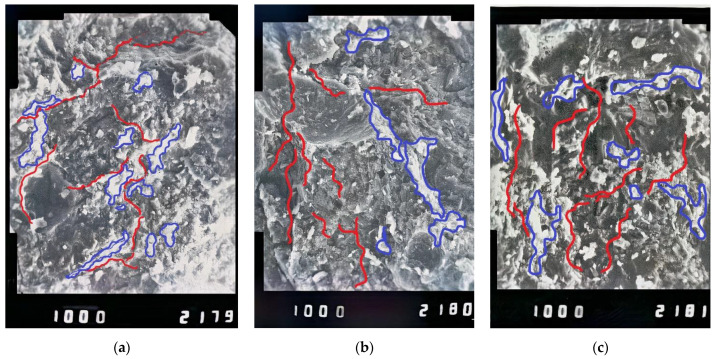
Microstructure of concrete after the action of sulfuric acid solution: (**a**) on the surface of the sample, (**b**) at a distance of 4–6 mm from the surface and (**c**) at a distance of 10 mm (cracks are marked with red, precipitates and ettringite crystals are marked with blue).

After the destruction of concrete prisms subjected to the impact of sulfuric acid, the cut of the prisms in the zone adjacent to the place of concrete destruction was performed with a diamond saw (Figure 15). As a result, with the use of an X-ray electron microscope with magnification of 1000 times, in the outer layer, corrosion products were detected. The thickness of this layer was 2.6–3.1 mm for samples of all series. The inner layers of concrete had an intact structure, and no corrosion processes were detected (Figure 15 and Figure 16).

**Figure 17:**
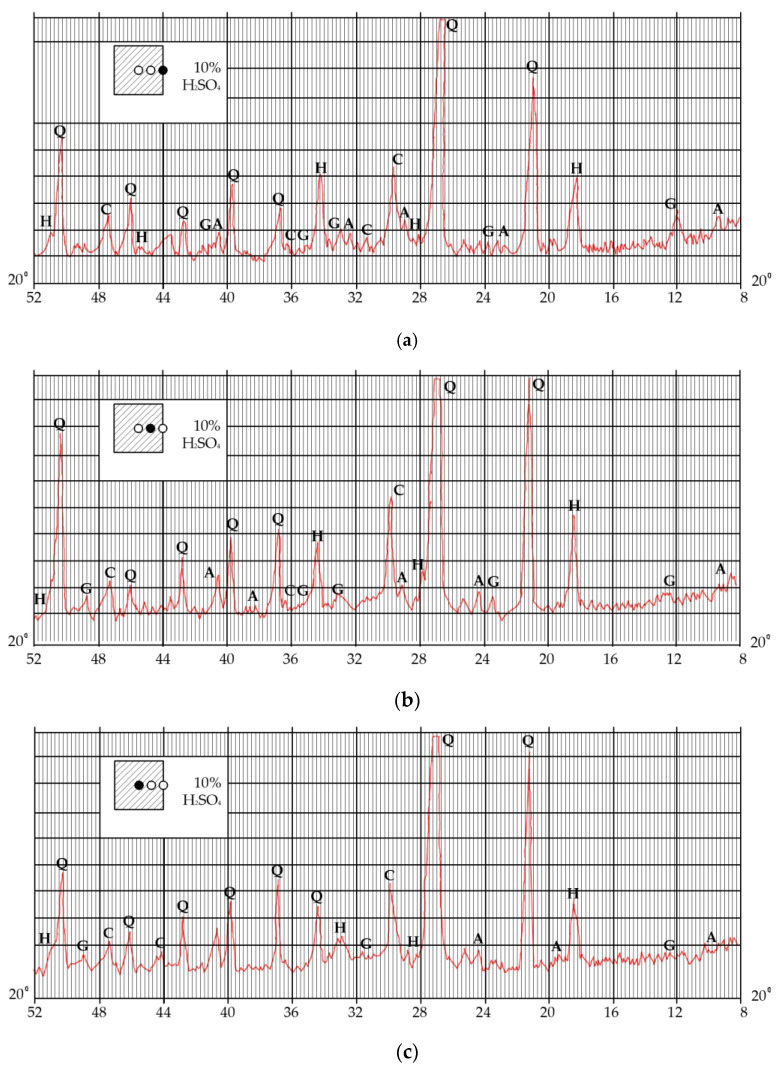
Diffractogram of X-ray phase analysis of concrete after the action of the H_2_SO_4_ solution: (**a**) near the surface, (**b**) at a distance of 4–6 mm from the surface and (**c**) at a distance of 10 mm from the surface (Q—quartz (SiO_2_), C—calcid (CaCO_3_), H—calcium hydroxide (Ca(OH)_2_), G—gypsum (CaSO_4_∙2H_2_O), A—calcium aluminohydrochloride (3CaO∙Al_2_O_3_∙3CaSO_4_∙32H_2_O)).

These results could also be confirmed by X-ray phase analysis (Figure 17). Studies performed with the use of an electron microscope showed that microcracks were detected in the contact layer. Comparison of the microstructure of intact and corrosion-damaged concrete layers shows that microcracks were caused by the corrosion of concrete. Due to the impact of acid, corrosion products were formed, such as gypsum, which precipitates and ettringite crystals, and which is confirmed by X-ray analysis of cement stone. It is known that during the process of formation of ettringite crystals, their volume increases by 2–2.5 times, which leads to additional destruction of the concrete. This effect was confirmed by the presence of microcracks in photographs taken under the microscope with magnification of 1000 times.

Analysis of the results of experimental studies shows that the destruction of concrete prisms in sulfuric acid was due to concrete corrosion. As a result of concrete corrosion, a contact layer with lower physical and mechanical characteristics was formed. The thickness of the contact layer for samples of series 2 was 1.5 times smaller than for series 1. This could be explained by the use of chemical additives in samples of series 2 and more careful selection of concrete components. In the contact layer of the prism concrete, the presence of the corrosion product ettringite was detected, which causes the formation of microcracks. These microcracks become stress concentrators in concrete. This explains the decrease in the concrete strength during long-term loading, which could be taken into account by the coefficient of working conditions of concrete in an aggressive environment.

## 4. Conclusions

Experimental research conducted in this work detailed the study of stress–strain of concrete samples, subjected to simultaneous action of aggressive impact and constant loading. As a result, changes of cross-section dimensions of samples were recorded, “*σ_c_-ε*” diagrams were obtained and specifics of stress and deformation changes over time were identified. These results bring us to the conclusion that reasons for the destruction of concrete prisms are the presence of corrosion damages in the concrete and the cross-section reduction. For different series of samples, different exposure times in an aggressive environment were required for destruction: for series 1–106 days, for series 2–79 days, for series 3–56 days and for series 4–89 days. Decreases of cross-sectional sizes were also controlled and recorded. Namely, the relative reduction of the area for samples of series 1 was on average 63–65%, whereas for series 2, 3 and 4 this number reached 46–50%, 34–37% and 4–51%, respectively. Therefore, the load level has a significant influence on the residual load-bearing capacity of samples, subjected to corrosion. In addition, ultimate loads were calculated with the use of the theoretical approach and compared with experimental results. As the theoretical values were on average 15–17% higher than the obtained experimental values, it could be assumed that the stresses in the concrete at the moment of destruction were less than the value of the concrete prism strength. Therefore, exhaustion of the load-bearing capacity of concrete samples is associated not only with the cross-section decrease, but also with the reduction of material strength characteristics.

The ultimate deformations of concrete with corrosion damages, which correspond to the prismatic strength of concrete in comparison with concrete without corrosion damage, were lower by 18% for material with $f_{ck}=41$ MPa and by 11.8% for samples with $f_{ck}=53.6$ MPa.

In order to obtain reliable information about the corrosion mechanisms, which cause a reduction of the physico-mechanical characteristics of the concrete, X-ray phase analysis with the use of an electron microscope was performed. It was identified that along the section contour, a partially degraded layer of concrete was formed with a thickness of 1.5–3.7 mm, with corrosive microcracks and corrosion products. Comparison of the microstructure of intact and corrosion-damaged concrete layers enabled to indicate gypsum and ettringite crystals, which are corrosion products and are the reason for additional initial stresses in concrete.

It could be concluded that according to the results of the experimental research, deterioration of concrete in aggressive environments is associated with two significant aspects: the decrease of cross-sectional dimensions and changes of physico-mechanical characteristics of the material. It was identified that theoretical algorithms for the calculation of the load-bearing capacity of damaged concrete elements overestimate strength characteristics and do not fully represent the corrosion mechanisms. It was also shown that the intensity of exhaustion of samples’ strength to a large extent depends on the level of load applied and environmental conditions.

Findings of this study are important and need to be taken into consideration when the sustainability of corroded concrete structures is evaluated.

## Figures and Tables

**Table 1:** Mixing composition of the concrete.

Component	Amount, kg/m^3^	% of Mass
Cement	500	21.6
Quartz sand	505	21.9
Granite crushed stone	1135	49.2
Water	160	6.9
Super-plasticizer “Chrysofluid”	6	0.26
Air-absorbing additive “Chrysoair”	0.17	0.01

**Table 2:** Characteristics of the cement.

Characteristics	M400 Grade	M500 Grade
Marking according to DSTU BV 2.7–46: 2010	CEM I 32.5 N	CEM I 42.5 N
The material composition of cement by weight, %	Portland cement clinker 100%	
Specific gravity, g/cc	3.11	3.15
Compressive strength, MPa		
2nd day	26.0	36.0
28th day	48.0	56.0
Setting time, minutes		
initial	160	140
final	240	220
Fineness of Cement, %	7.3	8.5

**Table 3:** Strength of concrete prisms subjected to simultaneous action of loading and the aggressive environment.

Series	Marking	Concrete Strength, *f_ck_*, MPa	Residual Cross-Sectional Area, *S*, cm^2^	Flexibility, λ	γ_m_	Ultimate Load, N_ult_, kN		Difference, %
						Theoretical (Eurocode 2)	Experimental	
Series 1	PL-1.1c	41.0	37.8	6.5	0.92	126.5	104.6	17.3%
	PL-1.2c	41.0	36.9	6.7		123.9	104.3	15.6%
	PL-1.3c	41.0	35.5	6.8		118.7	103.5	12.8%
Series 2	PL-2.1c	41.0	54.8	5.4		185.3	149.3	19.4%
	PL-2.2c	41.0	51.3	5.6		171.7	147.8	13.9%
	PL-2.3c	41.0	53.3	5.5		178.3	143.5	19.5%
Series 3	PL-3.1c	41.0	67.0	4.9		226.7	188.2	17.0%
	PL-3.2c	41.0	65.6	5.0		220.1	184.5	16.2%
	PL-3.3c	41.0	64.1	5.0		214.5	188.1	12.3%
Series 4	PL-4.3c	53.6	51.8	5.6		230.1	190.1	17.4%
	PL-4.4c	53.6	49.0	5.7		217.5	191.0	12.2%
	PL-4.5c	53.6	51.1	5.6		226.9	190.5	16.4%

**Table 4:** Experimental and theoretical values of the ratio N_ult_/S and measurement errors.

Series	Marking	Ratio N_ult_/S, kN/cm^2^		Difference, %	Measurement Error	
		Theoretical	Experimental		Instrumental	Total, %
Series 1	PL-1.1c	3.35	2.77	17.3%	up to 2 divisions (2%)	2.4%
	PL-1.2c	3.35	2.82	15.6%		0.7%
	PL-1.3c	3.34	2.92	12.8%		3%
Series 2	PL-2.1c	3.38	2.72	19.4%		1.7%
	PL-2.2c	3.35	2.88	13.9%		4.2%
	PL-2.3c	3.35	2.69	19.5%		2.7%
Series 3	PL-3.1c	3.38	2.81	17.0%		1.4%
	PL-3.2c	3.36	2.81	16.2%		1.4%
	PL-3.3c	3.35	2.93	12.3%		2.8%
Series 4	PL-4.3c	4.44	3.67	17.4%		2.5%
	PL-4.4c	4.44	3.89	12.2%		3.4%
	PL-4.5c	4.44	3.73	16.4%		1%

## Data Availability

The data presented in this study are available from the corresponding author upon request.
